# Supplementary material for: Augmented curation of clinical notes from a massive EHR system reveals symptoms of impending COVID-19 diagnosis
Source: eLife. 2020 Jul 7;9:e58227. doi: 10.7554/eLife.58227 (PMC7410498; doi:10.7554/eLife.58227)
Supplement: Supplementary file 1. — (A) Enrichment of diagnosis codes amongst COVIDpos patients in the week preceding PCR testing. (B) Enrichment of diagnosis codes amongst COVIDneg patients in the week preceding PCR testing. (C) Symptoms and their synonyms used for the EHR analysis. (D) Pairwise analysis of symptoms in the COVIDpos and COVIDneg cohorts. The pairwise symptom combinations with BH-corrected p-value<0.01 are summarized. (E) Patients with at least one clinical note over time. (F) SciBERT vs. BioClinicalBERT Phenotype Sentiment Model Performance on 18,490 sentences. (G) Model Performance Trained on 18,490 Sentences Containing 250 Different Cardiovascular, Pulmonary, and Metabolic Phenotype. (H) Model Performance Trained on 21,678 Sentences Containing 250 Different Cardiovascular, Pulmonary, and Metabolic Phenotypes and Expanded to Include 26 COVID-related Symptoms. (I) Synonym classification model performance [file elife-58227-supp1.docx]

**Supplementary file 1**

**Supplementary file 1A. Enrichment of diagnosis codes amongst COVID_pos_ patients in the week preceding PCR testing**

| **Diagnosis Code** | **Percentage of COVID_pos_ Patients** |
| --- | --- |
| Acute upper respiratory infection, unspecified | 20.67% |
| Cough | 5.45% |
| Other nonspecific abnormal finding of lung field | 1.95% |
| Fever, unspecified | 1.91% |
| Shortness of breath | 1.91% |
| Contact with and (suspected) exposure to other viral communicable diseases | 1.69% |
| Abnormal electrocardiogram (ECG) (EKG) | 1.23% |
| Pneumonia, unspecified organism | 1.14% |
| Pneumonia | 0.97% |
| Other viral pneumonia | 0.84% |
| Viral infection, unspecified | 0.81% |
| Personal history of nicotine dependence | 0.81% |
| Essential (primary) hypertension | 0.81% |
| Hyperlipidemia, unspecified | 0.76% |
| Acute respiratory failure with hypoxia | 0.67% |
| Hypokalemia | 0.63% |
| Shortness of Breath | 0.63% |
| Acute Respiratory Failure with Hypoxia | 0.63% |
| Chest pain, unspecified | 0.63% |
| Type 2 diabetes mellitus without complication | 0.55% |

**Supplementary file 1B. Enrichment of diagnosis codes amongst COVID_neg_ patients in week preceding PCR testing**

| **Diagnosis Code** | **Percentage of COVID_neg_ Patients** |
| --- | --- |
| Acute upper respiratory infection, unspecified | 26.40% |
| Cough | 2.81% |
| Shortness of breath | 2.41% |
| Encounter for other preprocedural examination | 2.31% |
| Essential (primary) hypertension | 2.01% |
| Hyperlipidemia, unspecified | 2.01% |
| Abnormal electrocardiogram (ECG) (EKG) | 1.88% |
| Other nonspecific abnormal finding of lung field | 1.78% |
| Fever, unspecified | 1.48% |
| Encounter for screening for other viral diseases | 1.44% |
| Personal history of nicotine dependence | 1.31% |
| Contact with and (suspected) exposure to other viral communicable diseases | 1.26% |
| Encounter for observation for suspected exposure to other biological agents ruled out | 1.20% |
| Unspecified atrial fibrillation | 1.19% |
| Tachycardia, unspecified | 1.16% |
| Other long term (current) drug therapy | 1.11% |
| Gastro-esophageal reflux disease without esophagitis | 1.09% |
| Chest pain, unspecified | 1.02% |
| Type 2 diabetes mellitus without complications | 0.94% |
| Long term (current) use of anticoagulants | 0.93% |

**Supplementary file 1C. Symptoms and their synonyms used for the EHR analysis.**

| **Symptom/Finding** | **Synonyms/related entities identified in EHR** |
| --- | --- |
| Fever / chills | fever, fevers, chill, chills, tactile fever, felt warm, subjective fever |
| Altered or diminished sense of taste or smell | change in smell, lost her sense of smell and taste, bitter taste in his mouth, no sense of taste or smell, no sense of smell or taste, decrease in smell, decreased sense of taste, change in her sense of taste and smell, decrease in smell and taste, ageusia, change in taste, lost her sense of taste and smell, dysgeusia, everything smells and tastes terrible, anosmia, altered smell, loss of taste and smell, change in his sense of smell and taste, altered sense of taste and smell, decrease in taste and smell, decreased taste, bitter taste in her mouth, taste is altered, lost his sense of smell, decreased smell, altered taste, altered sense of smell and taste, change in taste and smell, lost his sense of smell and taste, change in her sense of smell and taste, everything tastes and smells terrible, lost his sense of taste and smell, lost her sense of smell, loss of smell and taste, decrease in taste, decrease taste, bitter taste, no smell or taste, no taste or smell, anosmia/dysgeusia, decrease smell, change in smell and taste, change in his sense of taste and smell |
| Diarrhea | loose stools, soft stools, watery diarrhea, soft stool, diarrhea, watery bm, vomiting diarrhea, loose stool, diarrhea vomiting |
| GI upset | abdominal pain, nausea, abdominal cramping, posttussive emesis, stomach ache, emesis, vomiting, nausea vomiting abdominal pain, stomach cramping, vomiting diarrhea, diarrhea vomiting |
| Wheezing | wheezing |
| Respiratory difficulty | increased oxygen demands, lower respiratory symptoms, tachypnea, tachypneic, labored breathing, dyspnea with walking, dyspnea on exertion, shortness of breath, sob with exercise, dyspnea, sob |
| Respiratory failure | Respiratory failure |
| Cough | cough, cough that is nonproductive, productive cough, cough np p, dry cough, cough productive, cough that is productive, nonproductive cough, coughing, non productive cough |
| Hemoptysis | hemoptysis, blood-tinged sputum, red-tinged sputum |
| Chest pain/pressure | chest congestion, chest tightness, pleuritic chest pain, chest heaviness, chest pain, tightness of the chest, chest discomfort |
| Congestion | sinus pressure, congestion, head congestion, stuffy nose, congested, nasal congestion, congestion rhinorrhea, facial pressure, sinus congestion |
| Rhinitis | rhinitis, itchy eyes and nose, runny nose, tickling in nose, sniffles, rhinorrhea, sneezing, congestion rhinorrhea |
| Myalgia/Arthralgia | body ache, myalgia, muscle aches, arthralgias, sore neck, sore muscles, muscle discomfort, joints became sore, body aches, aches and pains, achy joints, arthralgia, myalgias], Generalized symptoms: [weakness, feeling run down, cold, malaise, generalized weakness, influenza like symptoms, weak, feeling poorly |
| Generalized symptoms | cold, generalized weakness, malaise, weakness, weak, influenza like symptoms, feeling run down, activity change |
| Fatigue | fatigued, energy level is diminished, lethargy, fatigue, energy level is poor, activity change, sleeping more than usual, lethargic |
| Diaphoresis | diaphoretic, diaphoresis, night sweats, sweaty, sweating, sweats |
| Pharyngitis | scratchy throat, throat discomfort, tingly throat, throat irritation, sore throat |
| Headache | headache, HA, headaches, HA's, sinus headache |
| Dry mouth | dry mouth, xerostomia |
| Change in appetite/intake | decrease in appetite, diminished appetite, appetite is poor, appetite change, appetite is diminished, appetite has been fluctuating, poor appetite, decreased appetite, anorexia, not eating and drinking, little appetite, no appetite, not eating or drinking |
| Conjunctivitis | itchy eyes and nose, red eyes, watery eyes, pink eye, red eye, watery eyes with redness |
| Neuro | agitation, vision trouble, dizziness, confusion, delirium, agitated |
| Cardiac | palpitations, lightheadedness |
| Otitis | earache, ear ache, ear pain |
| Dermatitis | rash |

**Supplementary file 1D. Pairwise analysis of symptoms in the COVID*_pos_* and COVID*_neg_* cohorts.** The pairwise symptom combinations with BH-corrected p-value < 0.01 are summarized.

| **Symptom 1** | **Symptom 2** | **COVID+**  **Count (%)**  **(N=635)** | **COVID-**  **Count (%) (N=29859)** | **(COVID+)/(COVID-) Relative Ratio** | **Relative Ratio (95% CI)** | **Raw**  **p-value** | **BH-corrected p-value** |
| --- | --- | --- | --- | --- | --- | --- | --- |
| Fever / chills | Cough | 693 (29.9%) | 5341 (7.1%) | 4.19 | (3.92, 4.48) | 9.73E-224 | 1.34E-221 |
| Fever / chills | Headache | 290 (12.5%) | 2522 (3.4%) | 3.71 | (3.32, 4.17) | 3.89E-76 | 1.53E-74 |
| Cough | Headache | 290 (12.5%) | 2610 (3.5%) | 3.59 | (3.21, 4.03) | 4.26E-73 | 1.47E-71 |
| Fever / chills | Myalgia/Arthralgia | 250 (10.8%) | 2186 (2.9%) | 3.69 | (3.27, 4.19) | 6.12E-65 | 1.53E-63 |
| Cough | Myalgia/Arthralgia | 246 (10.6%) | 2239 (3.0%) | 3.55 | (3.14, 4.03) | 6.89E-61 | 1.58E-59 |
| Fever / chills | Rhinitis | 180 (7.8%) | 1435 (1.9%) | 4.05 | (3.5, 4.71) | 1.02E-51 | 1.87E-50 |
| Fever / chills | Congestion | 199 (8.6%) | 1943 (2.6%) | 3.31 | (2.88, 3.81) | 5.42E-45 | 7.48E-44 |
| Congestion | Rhinitis | 146 (6.3%) | 1293 (1.7%) | 3.65 | (3.1, 4.32) | 2.41E-37 | 3.02E-36 |
| Cough | Rhinitis | 182 (7.9%) | 2044 (2.7%) | 2.88 | (2.49, 3.33) | 6.51E-34 | 7.48E-33 |
| Diarrhea | Cough | 185 (8.0%) | 2139 (2.9%) | 2.79 | (2.42, 3.23) | 5.46E-33 | 5.58E-32 |
| Rhinitis | Headache | 108 (4.7%) | 850 (1.1%) | 4.10 | (3.39, 5.01) | 9.15E-32 | 9.02E-31 |
| Myalgia/Arthralgia | Headache | 152 (6.6%) | 1647 (2.2%) | 2.98 | (2.55, 3.51) | 6.10E-30 | 5.81E-29 |
| Congestion | Headache | 121 (5.2%) | 1250 (1.7%) | 3.13 | (2.62, 3.76) | 1.16E-25 | 1.00E-24 |
| Rhinitis | Myalgia/Arthralgia | 72 (3.1%) | 606 (0.8%) | 3.84 | (3.04, 4.91) | 3.88E-20 | 2.98E-19 |
| Fever / chills | Cardiac | 81 (3.5%) | 756 (1.0%) | 3.46 | (2.78, 4.36) | 5.60E-20 | 4.07E-19 |
| Congestion | Myalgia/Arthralgia | 95 (4.1%) | 1030 (1.4%) | 2.98 | (2.44, 3.68) | 4.01E-19 | 2.76E-18 |

**Supplementary file 1E. Patients with at least one clinical note over time**

| **Relative Day** | **COVID+ patients** | **COVID- patients** |
| --- | --- | --- |
| -7 | 57 | 3601 |
| -6 | 70 | 3866 |
| -5 | 76 | 4269 |
| -4 | 90 | 4345 |
| -3 | 96 | 5047 |
| -2 | 105 | 6060 |
| -1 | 1892 | 54836 |
| 0 | 1593 | 36655 |

**Supplementary file 1F. SciBERT vs. BioClinicalBERT Phenotype Sentiment Model Performance on 18,490 sentences**

| **Model** | **SciBERT Micro-F1** | **BioClinicalBERT Micro-F1** |
| --- | --- | --- |
| Phenotype Sentiment Model | 0.939 | 0.932 |

**Supplementary file 1G. Model Performance Trained on 18,490 Sentences Containing 250 Different Cardiovascular, Pulmonary, and Metabolic Phenotypes**

Overall Accuracy (90%:10% train-test split): 0.936

| Label | Precision | Recall | F1-Score |
| --- | --- | --- | --- |
| Yes | 0.97 | 0.96 | 0.96 |
| No | 0.97 | 0.95 | 0.96 |
| Maybe | 0.87 | 0.83 | 0.85 |
| Other | 0.81 | 0.91 | 0.86 |

**Supplementary file 1H. Model Performance Trained on 21,678 Sentences Containing 250 Different Cardiovascular, Pulmonary, and Metabolic Phenotypes and Expanded to Include 26 COVID-related Symptoms**

Overall Accuracy (90%:10% train-test split): 0.940

| Label | Precision | Recall | F1-Score |
| --- | --- | --- | --- |
| Yes | 0.96 | 0.97 | 0.96 |
| No | 0.96 | 0.96 | 0.96 |
| Maybe | 0.89 | 0.80 | 0.84 |
| Other | 0.88 | 0.88 | 0.88 |

**Supplementary file 1I. Synonym classification model performance.**

| Symptom(s) | Total # sentences | % from template | Overall accuracy | True Positive Rate | False Positive Rate |
| --- | --- | --- | --- | --- | --- |
| *COMBINED* | *4001* | *10.2* | 0.951 | 0.967 | 0.061 |
| Fever / chills | 915 | 13.4 | 0.943 | 0.969 | 0.077 |
| Cough | 913 | 11.6 | 0.948 | 0.958 | 0.061 |
| Respiratory difficulty | 751 | 13.7 | 0.961 | 0.980 | 0.049 |
| Diarrhea | 440 | 17.3 | 0.940 | 0.954 | 0.052 |
| Respiratory failure | 202 | 0.0 | 0.990 | 1.000 | 0.667 |
| Headache | 199 | 0.0 | 0.942 | 0.967 | 0.075 |
| Fatigue | 198 | 0.0 | 0.984 | 0.988 | 0.063 |
| Altered or diminished sense of taste or smell | 166 | 0.0 | 0.856 | 0.822 | 0.077 |
| Myalgia/Arthralgia | 78 | 0.0 | 0.986 | 1.000 | 0.037 |
| Dysuria | 69 | 0.0 | 0.956 | 0.929 | 0.019 |
| Change in appetite/intake | 67 | 0.0 | 1.000 | 1.000 | 0.000 |
| Diaphoresis | 3 | 0.0 | 1.000 | 1.000 | 0.000 |
